# Insights into high-efficiency lignocellulolytic enzyme production by *Penicillium oxalicum* GZ-2 induced by a complex substrate

**DOI:** 10.1186/s13068-014-0162-2

**Published:** 2014-11-18

**Authors:** Hanpeng Liao, Shuixian Li, Zhong Wei, Qirong Shen, Yangchun Xu

**Affiliations:** National Enginnering Research Center for Organic-based Fertilizers, Jiangsu Collaborative Innovation Center for Solid Organic Waste Utilization, College of Resources and Environmental Science, Nanjing Agricultural University, Nanjing, 210095 China

**Keywords:** Secretome, *Penicillium oxalicum*, Cellulose and xylan, Lignocellulolytic enzyme, Gene expression

## Abstract

**Background:**

Agricultural residue is more efficient than purified cellulose at inducing lignocellulolytic enzyme production in *Penicillium oxalicum* GZ-2, but in *Trichoderma reesei* RUT-C30, cellulose induces a more efficient response. To understand the reasons, we designed an artificially simulated plant biomass (cellulose plus xylan) to study the roles and relationships of each component in the production of lignocellulolytic enzymes by *P. oxalicum* GZ-2.

**Results:**

The changes in lignocellulolytic enzyme activity, gene expression involving (hemi)cellulolytic enzymes, and the secretome of cultures grown on Avicel (A), xylan (X), or a mixture of both (AX) were studied. The addition of xylan to the cellulose culture did not affect fungal growth but significantly increased the activity of cellulase and hemicellulase. In the AX treatment, the transcripts of cellulase genes (*egl1*, *egl2*, *egl3*, *sow*, and *cbh2*) and hemicellulase genes (*xyl3* and *xyl4*) were significantly upregulated (*P* <0.05). The proportion of biomass-degrading proteins in the secretome was altered; in particular, the percentage of cellulases and hemicellulases was increased. The percentage of cellulases and hemicellulases in the AX secretome increased from 4.5% and 7.6% to 10.3% and 21.8%, respectively, compared to the secretome of the A treatment. Cellobiohydrolase II (encoded by *cbh2*) and xylanase II (encoded by *xyl2*) were the main proteins in the secretome, and their corresponding genes (*cbh2* and *xyl2*) were transcripted at the highest levels among the cellulolytic and xylanolytic genes. Several important proteins such as swollenin, cellobiohydrolase, and endo-beta-1,4-xylanase were only induced by AX. Bray-Curtis similarity indices, a dendrogram analysis, and a diversity index all demonstrated that the secretome produced by *P. oxalicum* GZ-2 depended on the substrate and that strain GZ-2 directionally adjusted the compositions of lignocellulolytic enzymes in its secretome to preferably degrade a complex substrate.

**Conclusion:**

The addition of xylan to the cellulose medium not only induces more hemicellulases but also strongly activates cellulase production. The proportion of the biomass-degrading proteins in the secretome was altered significantly, with the proportion of cellulases and hemicellulases especially increased. Xylan and cellulose have positively synergistic effects, and they play a key role in the induction of highly efficient lignocellulolytic enzymes.

**Electronic supplementary material:**

The online version of this article (doi:10.1186/s13068-014-0162-2) contains supplementary material, which is available to authorized users.

## Background

With the exhaustion of fossil fuels and the increasing global demand for fuel, the enzymatic conversion of lignocellulosic feedstocks into fermentable sugars has become an attractive alternative for clean and sustainable fuel production. Fungi are the major sources of lignocellulolytic enzymes [1]. The lignocellulolytic enzymes used in enzymatic conversion mainly include cellulases and hemicellulases, which convert lignocellulolytic biomass into fermentable sugars. Although some progress has been made in enzyme production and in the enzymatic saccharification of lignocellulosic feedstocks, high cost is still the bottleneck hindering the industry’s development [2].

Over the past decades, many high-production cellulolytic fungi have been isolated and reported [3,4]. However, there have been relatively few reports concerning the induction and repression mechanisms of lignocellulolytic enzymes on complex substrates. High-yield lignocellulolytic enzyme production requires a corresponding substance as an inducer for the fungi. In general, cellulose is an effective inductive substrate for the production of cellulase by many filamentous fungi such as *Trichoderma* spp., *Aspergillus* spp., and *Penicillium* spp. Cellulose itself cannot directly trigger the induction of lignocellulolytic enzymes because it is insoluble [5]. Soluble saccharides such as cellobiose, sophorose, lactose, sorbose, and galactose have been demonstrated to induce cellulase synthesis in *Trichoderma reesei* [6-9]. Hemicellulase is usually induced by hemicellulolytic polymers. Xylans are the most effective inducers for xylanase production. However, the specificities in the induction of cellulase and hemicellulase have not been well characterized when a complex substrate is used as the inducer. Although cellulose is a good substrate for inducing cellulases, other enzymes like xylanase are also produced [10]. *Trichoderma longibrachiatum* cultured on a mixture of lactose (0.8%) and xylan (0.2%) was found to result in significantly higher levels of both xylanase and cellulase than did cultures on either substrate alone [11]. These reports suggested that complicated interactions exist in the induction of these enzymes.

Agricultural wastes are renewable and abundant worldwide, and they are available for use as cheap feedstock to produce biomass-degrading enzymes. Applications of these enzymes to saccharify plant biomass for the production of bioethanol have been reported elsewhere [12-14]. Many reports have found that agricultural wastes are superior to pure cellulose at inducing biomass-degrading enzymes in many fungi [15,16]. However, a comprehensive understanding of why a complex substrate can induce more biomass-degrading enzymes and how fungi respond to complex lignocellulose during enzyme production is still lacking. The regulation and secretion features of *Trichoderma* and *Aspergillus* have been well studied and characterized [17-19]. Recently, researchers have investigated how fungi respond to plant biomass and the mechanism behind this response using the model strain *Aspergillus niger* [20-22]. Many researchers have previously investigated the secretome and transcriptome of *Trichoderma* spp. and *Aspergillus* spp. induced by different types of plant biomass [10,23-26]. However, the mechanisms of enzymatic induction in different fungi display clear similarities but also exhibit differences in the regulation of the expression of cellulase- and hemicellulase-encoding genes [27]. In our previous studies, the lignocellulolytic enzyme activity induced by agricultural waste was significantly higher than that induced by purified cellulose from *P. oxalicum* GZ-2. However, this feature did not exist in strain *T. reesei* RUT-C30 (data not published in our lab), suggesting that *P. oxalicum* GZ-2 possibly has different regulation and induction mechanisms for producing lignocellulolytic enzymes.

Understanding how the filamentous fungus *P. oxalicum* GZ-2 responds to plant biomass and induces an enzyme cocktail to degrade plant polymers may result in new strategies to improve the production of second-generation biofuels. The relationships and roles of each complex plant biomass component (such as cellulose and xylan) in inducing and regulating lignocellulose-degrading enzyme gene expression and protein profiles are still poorly understood. Therefore, an artificially mixed substrate containing a mixture of Avicel and xylan was designed to simulate plant biomass in this study. The objective of this work was to investigate how inducible hydrolytic enzymes respond to cellulose and xylan and their relationships and roles in protein expression along with the activity of lignocellulolytic enzymes.

## Results

### Enzymatic activities induced by different substrates

**Figure 1:**
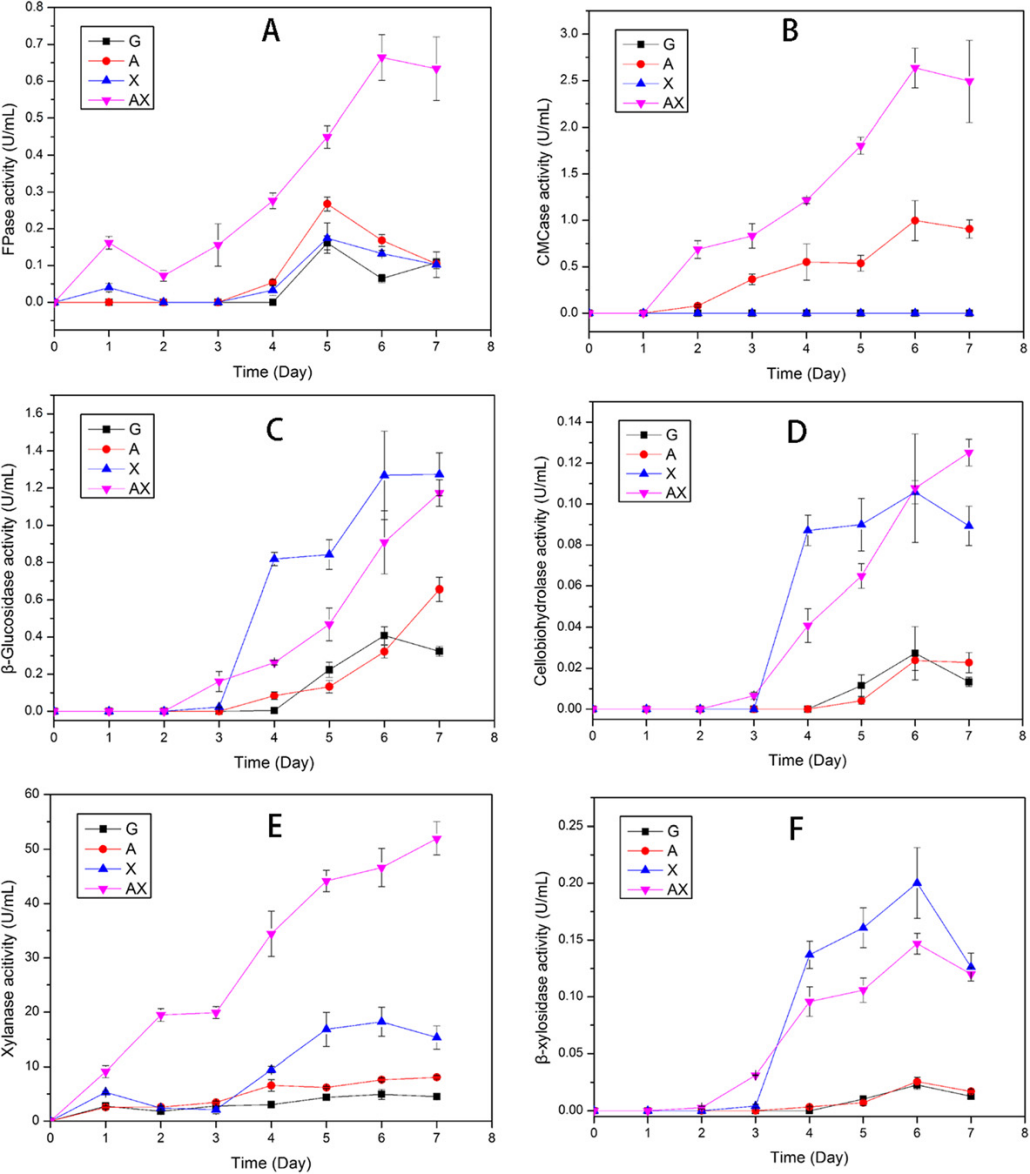
**Lignocellulolytic enzyme activities in the extracellular culture supernatant of** ***P. oxalicum*** **GZ-2 in the presence of different substrates.** Time courses of the enzyme activities of the various culture supernatants from *P. oxalicum* GZ-2 were determined during submerged fermentation for 7 days at 30°C. The activities of FPase, CMCase, beta-glucosidase, cellobiohydrolase, xylanase, and beta-xylosidase are listed in **A**, **B**, **C**, **D**, **E**, and **F**, respectively. The error bars indicate the standard deviation of three replicates.

The production of various enzymes by *P. oxalicum* GZ-2 was evaluated in a time course when glucose (G), Avicel (A), xylan (X), and a mixture of Avicel and xylan (AX) were used as the sole carbon source, and the results are shown in Figure 1. The highest activities of FPase, CMCase, and xylanase were obtained in the AX treatment, and the enzymatic activities of beta-glucosidase, beta-xylosidase, and cellobiohydrolase induced by AX were at the intermediate level. These enzymatic activities were the lowest in the G treatment. On day 7, the xylanase activity in the AX treatment was 3.5 and 6.5 times higher than that in the X and A treatments, respectively. The FPase, CMCase, and xylanase activities of *T. reesei* RUT-C30 are shown in Additional file 1: Figure S1. As expected, A and X were the best substrates for cellulase and xylanase production, respectively; the addition of xylan to the cellulose medium significantly decreased the FPase activity but had little effect on the xylanase activity (Additional file 1: Figure S1A).

To determine whether the increase in enzymatic activity by AX was due to an increase in fungal biomass from *P. oxalicum* GZ-2, we examined the growth behavior of *P. oxalicum* GZ-2 on various substrates. As shown in Additional file 2: Figure S2A, the growth of *P. oxalicum* GZ-2 on AX occurred at a slightly faster rate but with no significant difference before the third day. The fungal cells on A always increased with time and peaked at the end of the fermentation. The protein concentrations in the four substrates were determined and are shown in Additional file 2: Figure S2B. The maximum protein content (2.2 mg/mL) was found in the AX treatment during the later period of fermentation.

### Protein profiles in the culture supernatant

**Figure 2:**
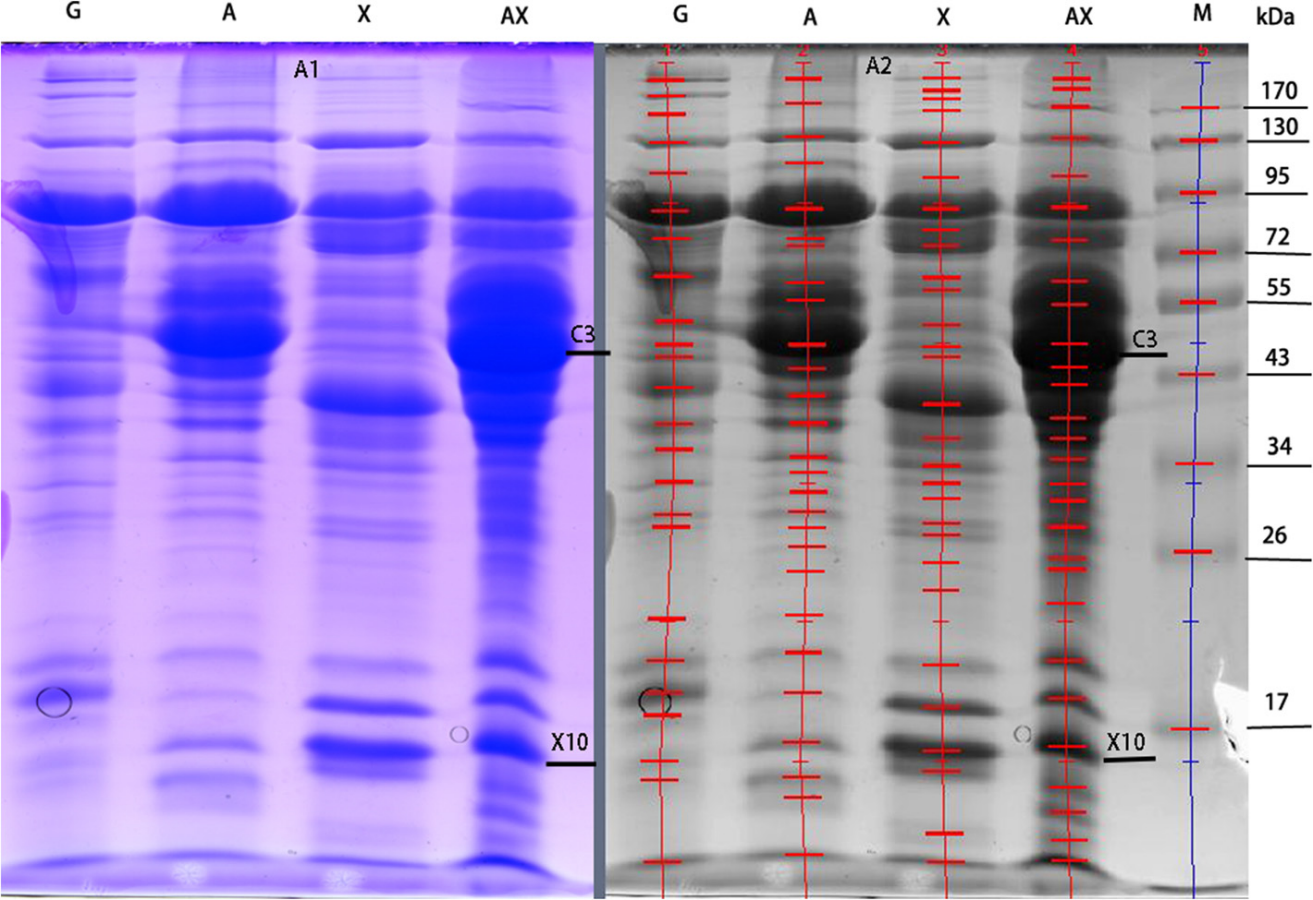
**Protein profile by SDS-PAGE (A1) as induced by the different substrates and band analysis using Quantity One (A2).** Each lane was loaded with approximately 100 μg of protein. Lane M: protein molecular weight markers.

**Figure 3:**
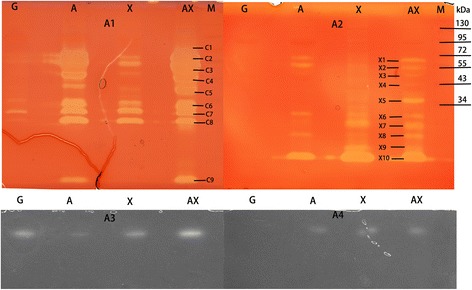
**Zymographic analysis of the expression of lignocellulolytic enzymes.** Zymographic analysis of the expression of endoglucanase **(A1)**, xylanase **(A2)**, beta-glucosidase **(A3)**, and cellobiohydrolase **(A4)** in the culture supernatant from *P. oxalicum* GZ-2 grown on different substrates. Lane M: protein molecular weight markers.

The protein profile by SDS-PAGE is shown in Figure 2A1. Automatic detection of the bands using Quantity One (Bio-Rad, USA) is shown in Figure 2A2. More protein bands were detected in the A, X, and AX lanes than in the G lane. Zymographic analysis demonstrated that more than eight, five, and nine protein bands with cellulase activity were observed in the A, X, and AX lanes, respectively (Figure 3A1). In the G lane, there were only three weak bands. As shown in Figure 3A1, the lanes AX and A have the same number of CMCase-active bands, but the pale-red hydrolysis zones of lane AX are clearly larger than those of lane A. In comparison with lane X, lane AX showed not only more protein bands but also clearly increased abundance of the protein bands. The bands from lane AX were excised, trypsin-digested, and further identified using matrix-assisted laser desorption/ionization time-of-flight tandem mass spectrometry (MALDI-TOF-MS/MS). The results in Table 1 indicate that most of the identified proteins are cellulases but also have other glycoside hydrolases. The hemicellulolytic profile of enzymes with xylanase activity analyzed by zymogram is shown in Figure 3A2. Similar to the cellulase zymogram, the greatest number of xylanase-active bands (ten bands) was observed in the AX lane, whereas no bands were found in the G lane. The MALDI-TOF-MS/MS identification results suggest that abundant hemicellulases such as putative endo-beta-1,4-xylanases, beta-1,4-mannanase, and alpha-L-arabinofuranosidase were found. We used in-gel activity assays to detect beta-glucosidase and cellobiohydrolase activity, and the results are shown in Figure 3A3,A4. In four lanes, one band showed beta-glucosidase activity in each lane, but the hydrolysis zone of the AX lane was the brightest. For cellobiohydrolase, one band was detected in each lane except for lane G.

**Table 1:** **Zymogram bands identification analysis using MALDI-TOF-MS/MS**

**Denotation**	**Predicted protein function**	**Family**	**Signal peptides**	**Protein score**	**Accession number**
C1	Exo-beta-1,3-glucanase	Pectate_lyase_3	Y	138	525586927
C2	Alpha-amylase Amy13A	Alpha-amylase	Y	51	525580015
	Glucoamylase Amy15A	GH15	Y	60	525588203
C3	Cellobiohydrolase CBHI/Cel7A-2	GH7	Y	143	525586734
	Swollenin	nd	Y	41	525580909
	Exo-beta-1,3-glucanase	GH17	Y	25	525581916
C4	Cellobiohydrolase Cel6A	GH6	Y	39	525585914
	Beta-1,4-mannanase	GH5	Y	80	525584819
C5	Endo-beta-1,4-glucanase	GH5	Y	231	525588012
C6	Hypothetical protein PDE_07927	nd	Y	57	525586716
C7	Hypothetical protein PDE_06814	nd	Y	105	525585606
C8	Endo-beta-1,4-xylanase	GH11	Y	145	525580908
C9	nd	nd	nd	nd	nd
X1	Glucoamylase Amy15A	GH15	Y	47	525588203
*X*2	Cellobiohydrolase CBHI/Cel7A-2	GH7	Y	150	525586734
	Endo-beta-1,4-xylanase	GH30	Y	103	525578833
	Exo-beta-1,3-glucanase	GH17	Y	25	525581916
X3	Cellobiohydrolase Cel6A	GH6	Y	39	525585914
X4	Beta-1,4-mannanase	GH5	Y	143	525584819
	Endo-beta-1,4-glucanase	GH5	Y	121	525588012
	Hypothetical protein PDE_07927	nd	Y	163	525586716
X5	Alpha-L-arabinofuranosidase	GH62	Y	193	525578835
X6	Beta-1,3-glucanosyltransferase	GH17	Y	28	525579431
X7	Endo-beta-1,4-xylanase	GH11	Y	144	525580908
X8	Lysozyme	GH25	Y	42	525582100
X9	Endo-beta-1,4-xylanase	GH11	Y	173	525583278
X10	Endo-beta-1,4-xylanase	GH11	Y	93	525583278

*nd* not detected; *Y* means with signal peptides; *GH* glycoside hydrolase.

### Transcript levels of lignocellulose-degrading genes in different substrates

**Figure 4:**
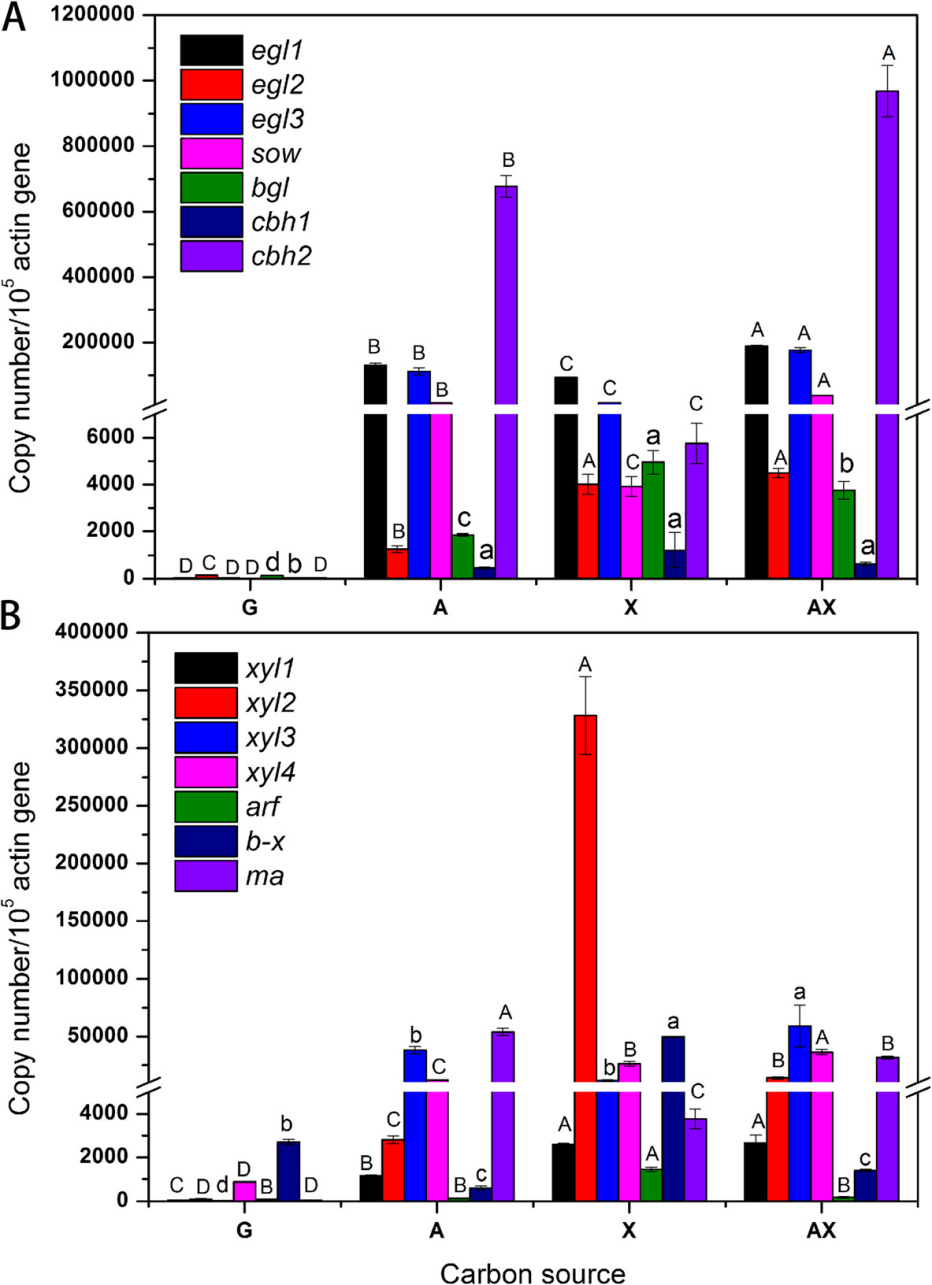
**Cellulolytic (A) and hemicellulolytic (B) enzyme gene expression levels of** ***P. oxalicum*** **GZ-2 grown on different substrates for 48 h.** The error bars indicate the standard deviation of three replicates. Values in different treatments followed by the same letter are not significantly different according to Tukey’s honest significant difference (HSD) test (capital letters indicate *P* <0.01; lowercase letters indicate *P* <0.05).

The transcript levels of lignocellulose-degrading genes in *P. oxalicum* GZ-2 under different substrate treatments are shown in Figure 4. The transcript profiles were determined on the second day because the growth of fungus GZ-2 nearly stopped after day 3. In the four substrate treatments, the studied genes encoding cellulose-degrading enzymes were transcribed at various levels (Figure 4A). The genes were transcribed to a significantly higher level under the A, X, and AX treatments than under the G treatment. The level of the *cbh2* transcript was significantly higher than those of the other enzyme-encoding genes in all the treatments. Similarly, significantly higher transcript levels of *egl1*, *egl2*, *egl3*, *sow*, and *cbh2* were detected in the AX treatment than in the A or X treatment (*P* <0.01). Unexpectedly, the highest level of the *bgl* transcript was observed in the X treatment. The expression level of *cbh1* was maintained at a low level in all treatments. A very low level of constitutive expression was observed for all the genes when G was used as a substrate.

All substrates (G, A, X, and AX) induced the expression of all the hemicellulolytic genes (*xyl1*, *xyl2*, *xyl3*, *xyl4*, *arf*, *b-x*, and *ma*) at various levels (Figure 4B). The most highly expressed gene among all the hemicellulolytic genes was *xyl2*. Expectedly, the expression of all the hemicellulolytic genes was significantly higher with the polymeric substrate than with glucose (at least *P* <0.05). Interestingly, the transcript levels of the genes *xyl3* and *xyl4* were induced significantly more by AX than by the other substrates (at least *P* <0.05). No transcript level difference of *xyl1* existed between the X and AX treatments, but it was significantly higher than that in the A treatment (*P* <0.01). The expression levels of *arf* and *b-x* were induced significantly more by X than by the other substrates. However, it is noteworthy that A was the substrate that most strongly induced the expression of *ma*.

### The secretome of *P. oxalicum* GZ-2 as induced by different substrates

**Figure 5:**
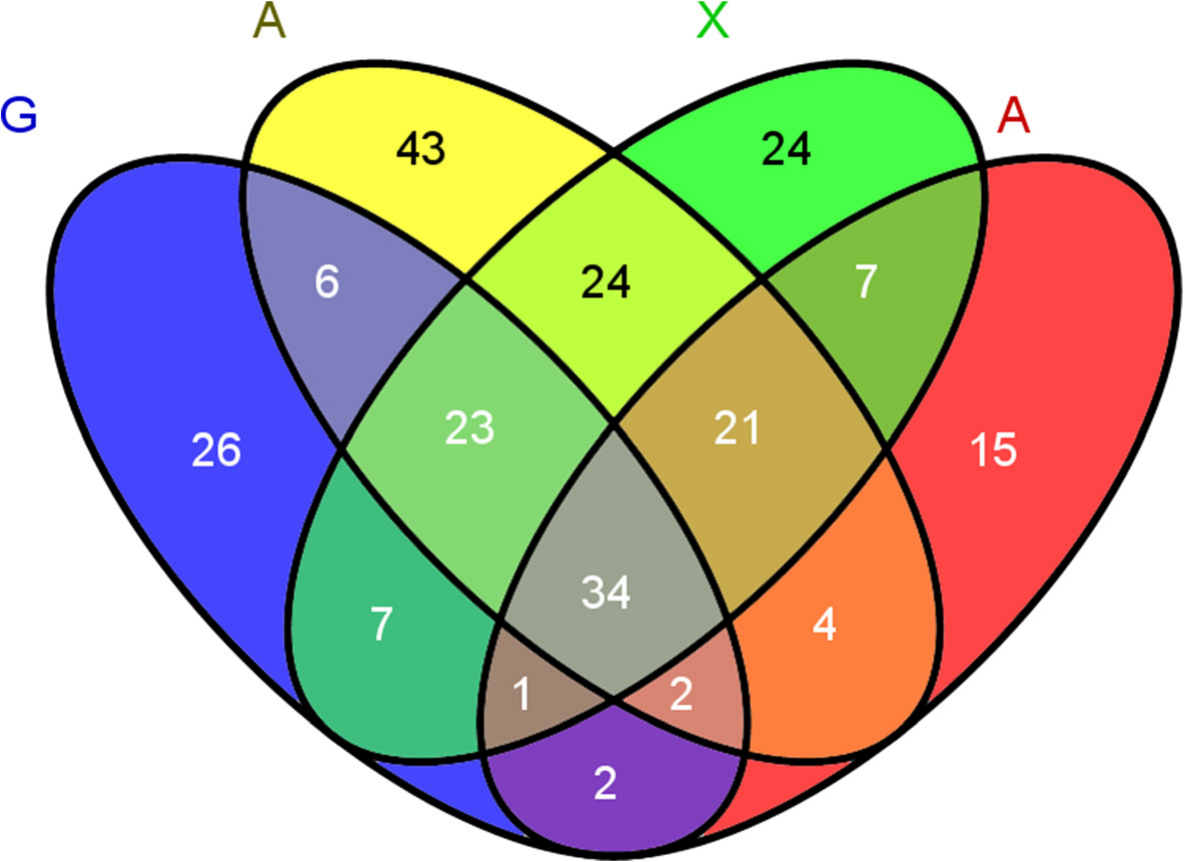
**Venn diagram representing unique and common proteins identified in the** ***P. oxalicum*** **GZ-2 secretome as induced by different substrates.** A total of 254 nonredundant protein groups were identified in the secretomes that resulted from growth in media containing G (glucose), A (Avicel), X (xylan), or AX (Avicel and xylan) using gel-free LC-MS/MS. The Venn diagram was prepared using Venny online tools.

**Figure 6:**
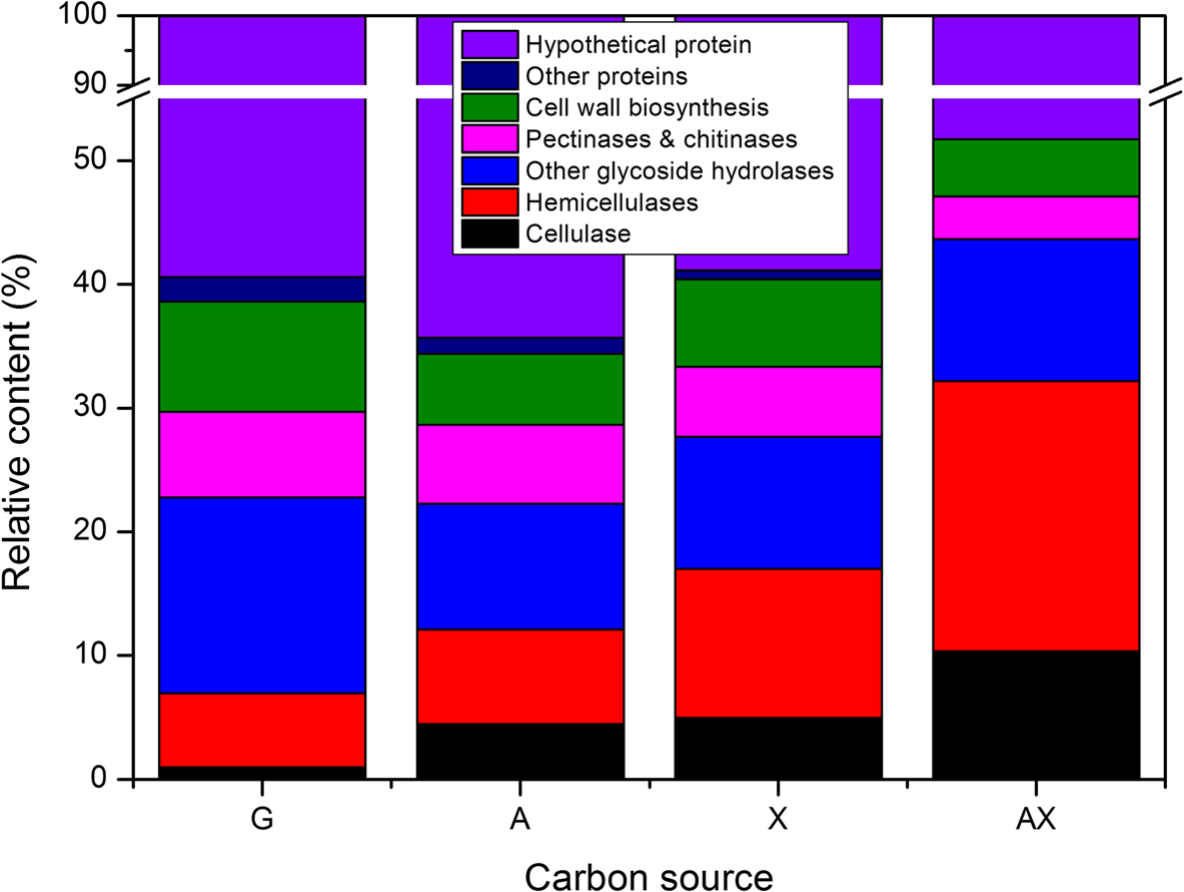
**Functional classification and relative proportion of the LC-MS/MS-identified proteins in the secretomes induced by the different substrates.**

**Figure 7:**
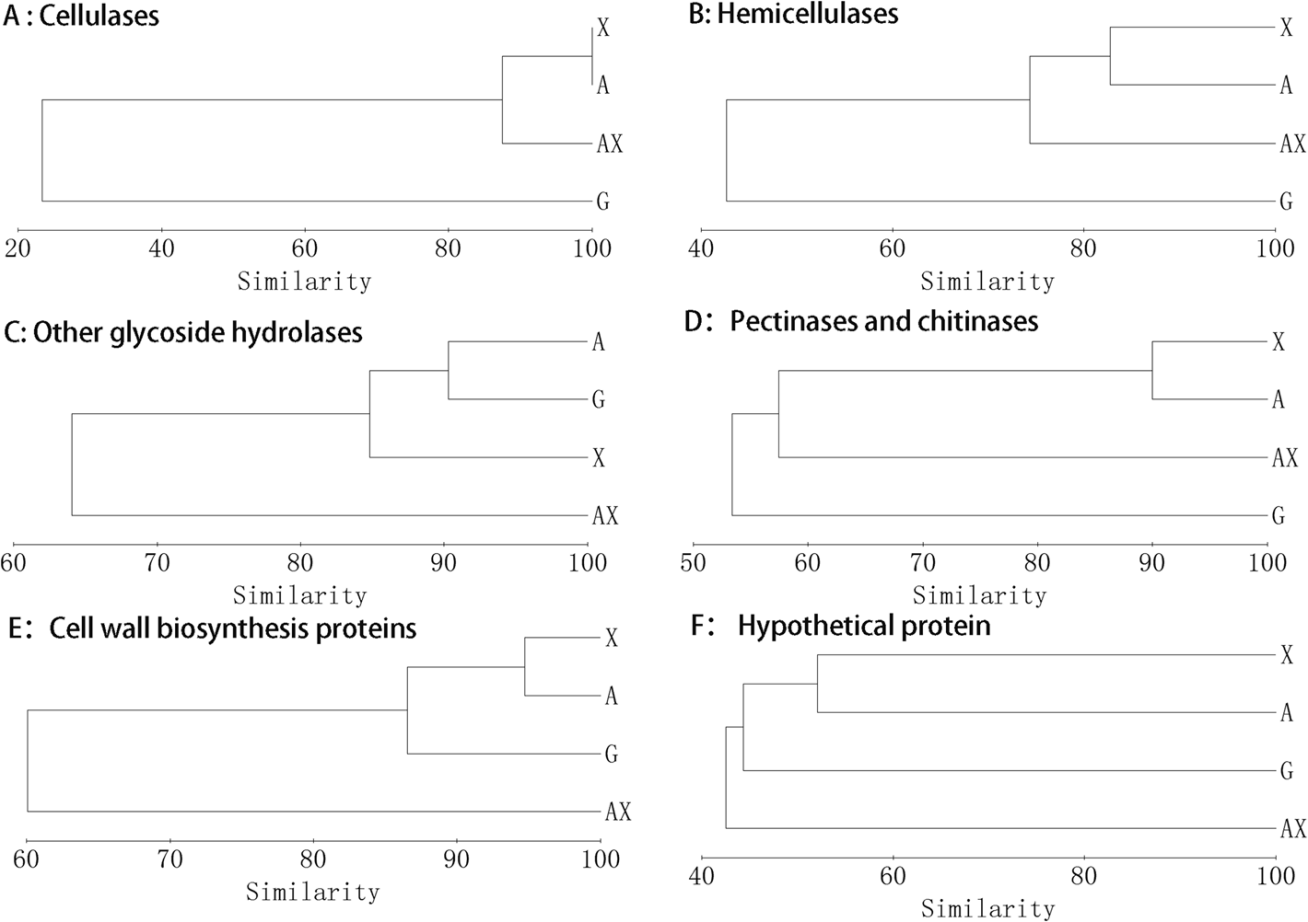
**Clustering analysis based on the detection/non-detection of individual proteins by LC-MS/MS in the secretome of** ***P. oxalicum*** **GZ-2 induced by different substrates.** The clustering analysis of cellulases **(A)**, hemicellulases **(B)**, other glycoside hydrolases **(C)**, pectinases and chitinases **(D)**, cell wall biosynthesis proteins **(E)**, and hypothetical protein **(F)** were performed based on the values of Bray-Curtis similarity.

Because the zymogram does not accurately show changes of a single protein, liquid chromatography-tandem mass spectrometry (LC-MS/MS) was used to analyze and identify the secretomes induced by different substrates. In the Venn diagram (Figure 5), 108 of the identified proteins (42.5%) exclusively existed in just one condition. A comparison of the four secretomes illustrates that 131 of the identified proteins (51.6%) were shared by two or three cultures. However, only 34 proteins were present in all four secretomes, corresponding to 13.4% of all the proteins (Additional file 3: Table S1). The proteins identified in the secretome of *P. oxalicum* GZ-2 grown on different substrates (G, A, X, and AX) are listed in Additional file 4: Table S2. As shown in Figure 6, the proteins identified in the secretomes were functionally grouped into cellulases, hemicellulases, other glycoside hydrolases, pectinases and chitinases, cell wall biosynthesis and metabolism proteins, hypothetical proteins, and other proteins. The percentage of cellulases was 1.0%, 4.5%, 5.0%, and 10.3%, respectively, in the G, A, X, and AX secretomes. The percentage of hemicellulases in the G, A, X, and AX secretomes was 5.9%, 7.6%, 12.1%, and 21.8%, respectively. The distributions of the molecular weights and isoelectric points of the identified proteins are shown in Additional file 5: Figure S3. The molecular weights of most of the identified proteins ranged from 15 to 150 kDa, and their isoelectric points ranged from 4.0 to 11.5. The similarity value among lignocellulose-degrading proteins (cellulases and hemicellulases) secreted on the A, X, and AX substrates, respectively, by the Bray-Curtis algorithm was >70% (Table 2). However, the similarity value was <50% between the lignocellulose-degrading proteins (cellulases and hemicellulases) induced by G and the other substrates. The clustering pattern of cellulases, hemicellulases, and other proteins is presented in Figure 7. The expression pattern of cellulases and hemicellulases induced by AX was clustered with A and X, while G formed another separate cluster (Figure 7A). However, the expression pattern of other glycoside hydrolases, cell wall biosynthesis metabolism proteins, and hypothetical proteins induced by G was clustered with A and X, and another separate cluster was formed by AX. The effects of the various substrates on the diversity of the functional proteins expressed by *P. oxalicum* GZ-2 were further evaluated using the Shannon-Wiener index (H) and the Simpson diversity index (D) (Additional file 6: Table S3). The H index of the identified proteins induced by G, A, X, and AX was 0.64, 0.63, 0.60, and 0.76, respectively. The D index of the identified proteins induced by G, A, X, and AX was 1.96, 1.98, 1.93, and 2.27, respectively.

**Table 2:** **Bray-Curtis similarity indices of the protein profile by** ***P. oxalicum*** **GZ-2 on different substrates**

**Cellulases**	**G**	**A**	**X**	**AX**
G	100			
A	25	100		
X	25	100	100	
AX	20	87.5	87.5	100
Hemicellulases				
G	100			
A	44.44	100		
X	43.48	82.76		
AX	40	70.97	77.78	100
Other glycoside hydrolases				
G	100			
A	90.32	100		
X	80	89.66	100	
AX	56	66.67	69.57	100
Pectinases and chitinases				
G	100			
A	55.56	100		
X	50	90	100	
AX	54.55	53.33	61.54	100
Cell wall biosynthesis metabolism proteins				
G	100			
A	88.89	100		
X	84.21	94.74	100	
AX	61.54	61.54	57.14	100
Hypothetical proteins				
G	100			
A	49.23	100		
X	39.46	52.07	100	
AX	40.43	46.55	40.6	100

A total of 101 proteins were identified when GZ-2 was grown in a medium containing 1% (w/v) G, and 26 proteins were only induced by G. Only one cellulase (beta-glucosidase BGL1, 525581542) was identified in the G culture. In the secretome obtained from the A culture, 157 proteins were identified, and 43 proteins were only induced by A. Three major types of cellulase (endoglucanase, beta-glucosidase, and cellobiohydrolase) and many hemicellulases were present in this secretome. A total of 141 proteins were identified, and 24 proteins exclusively existed in the X secretome. In the secretome of AX, 86 proteins were identified, 15 of which were only induced by AX. Two cellulases (swollenin and cellobiohydrolase) and four hemicellulases were specifically induced by AX.

### Expression and identification of cellulolytic proteins

Complete cellulose degradation requires three major cellulases: endo-1,4-beta-glucanase, exo-1,4-beta-glucanase, and beta-glucosidase. The greatest number of cellulose-degrading enzymes (nine cellulases) including three major cellulases, swollenin, and cellulose monooxygenase (Cel61A) were identified in the AX secretome. The proportion of cellulases in the AX secretome (10.3%) was more than twofold greater than that of the A (4.5%) or X (5.0%) secretome. The same number of cellulases (seven) was identified in the A and X secretomes. Only one cellulase was detected when *P. oxalicum* GZ-2 was grown on G. The comparative quantitative expression abundances of the cellulolytic proteins on the different substrates are presented in Table 3. The AX/A ratios of the putative beta-1,3-1,4-glucanase, cellulose monooxygenase Cel61A, and cellobiohydrolase Cel6A were 4.46, 2.12, and 7.15, respectively. The expression of most cellulases was strongly upregulated in the AX treatment compared to that in the X treatment. The abundance of most cellulases was more than 100-fold higher in the AX treatment than in the X treatment.

**Table 3:** **Label-free quantitative analysis of cellulolytic enzymes expression using SIEVE software**

**Accession**	**Predicted protein function**	**Signal peptides**	**Family**	**Ratio AX/A**	***P*** **-value**	**Ratio AX/X**	***P*** **-value**
525580909	Swollenin	Y	nd	nd	nd	nd	nd
525584244	Cellobiohydrolase	Y	GH7	nd	nd	nd	nd
525581542	Beta-glucosidase	Y	GH3	1.05	9.90E-20	82.65	2.29E-06
525581794	Beta-1,3-1,4-glucanase	Y	GH16	4.46	2.95E-08	0.65	0.9994203
525584431	Cellulose monooxygenase	Y	GH61	2.13	7.40E-07	1.14	0.8792061
525585914	Cellobiohydrolase	Y	GH6	7.15	8.39E-14	166.96	4.00E-11
525586734	Cellobiohydrolase	Y	GH7	0.80	9.90E-20	607.47	9.90E-20
525588012	Endo-beta-1,4-glucanase	Y	GH5	1.50	0.0002096	358.05	6.20E-12
525588754	Endo-beta-1,4-glucanase	Y	GH5	1.33	0.0011941	147.03	3.54E-08

*nd* not detected; Y means with signal peptides; *GH* glycoside hydrolase.

### Expression and identification of hemicellulases and glycoside hydrolases

The degradation of complex hemicelluloses requires several synergistic actions of different hemicellulases including xylanases, beta-xylosidase, beta-1,4-mannanase, acetyl xylan esterase, and alpha-L-arabinofuranosidase. These enzymes were present when *P. oxalicum* GZ-2 was grown on polymeric substrates (Additional file 4: Table S2). The numbers of identified proteins involved in hemicellulose degradation were 6, 12, 17, and 19 in the G, A, X, and AX treatments, respectively, and two putative alpha-L-arabinofuranosidases (525578834 and 525586375), a putative endo-beta-1,4-xylanase (525581488), and a putative acetyl xylan esterase (525588064) were only induced by AX. The AX/X ratios of the putative beta-1,4-mannanase (525584819), the putative endo-beta-1,4-xylanase (525578833), and the putative endo-beta-1,4-xylanase (525586882) were 4.9, 33.5, and 3.4, respectively. Various numbers of putative endo-beta-1,4-xylanases (5, 5, 4, and 1) were identified in the *P. oxalicum* GZ-2 secretomes induced by AX, X, A, and G, respectively. GH30 (previously classified into GH5) endo-beta-1,4-xylanase and GH3 beta-xylosidase are constitutively expressed using G as a substrate. The comparative expression abundances of the hemicellulolytic proteins cultured with various substrates are presented in Table 4. The AX/A ratios of the putative endo-beta-1,4-xylanase (525578833), the putative endo-beta-1,4-xylanase (525578833), the putative acetyl xylan esterase (525582983), the putative alpha-L-arabinofuranosidase (525584862), and the putative exo-beta-1,3-galactanase (525588065) were 2.9, 3.0, 9.1, 2.5, and 1.9, respectively. GH30 putative endo-beta-1,4-xylanase (525578833), GH11 putative endo-beta-1,4-xylanase (525580908), GH10 putative endo-beta-1,4-xylanase (525586882), putative alpha-L-arabinofuranosidase (525578835), and putative beta-1,4-mannanase (525584819) were upregulated when *P. oxalicum* GZ-2 was cultured with AX compared to X, and the AX/X ratios were 33.5, 1.4, 3.4, 2.4, and 4.9, respectively. Many other glycoside hydrolases involved in the hydrolysis of glycosidic bonds were identified in the GZ-2 secretome. Although no starch is present in any of the substrates, amylase and glucoamylase were identified in the A, X, and AX secretomes.

**Table 4:** **Label-free quantitative analysis of hemicellulolytic enzymes expression by SIEVE**

**Accession**	**Predicted protein function**	**Signal peptides**	**Family**	**Ratio AX/A**	***P*** **-value**	**Ratio AX/X**	***P*** **-value**
525582983	Acetyl xylan esterase	Y	Cutinase	9.07	9.90E-20	0.05	0.5983891
525578835	Alpha-L-arabinofuranosidase	Y	GH62	nd	nd	2.43	9.90E-20
525584862	Alpha-L-arabinofuranosidase	Y	GH43	2.49	0.0027907	0.11	0.0266966
525584819	Beta-1,4-mannanase	Y	GH5	0.36	9.90E-20	4.94	1.75E-11
525578868	Beta-xylosidase	Y	GH3	0.09	9.90E-20	0.07	1.59E-05
525588496	Beta-xylosidase	Y	GH3	0.27	1.15E-05	nd	nd
525578833	Endo-beta-1,4-xylanase	Y	GH30	2.99	9.90E-20	33.54	3.86E-12
525580908	Endo-beta-1,4-xylanase	Y	GH11	nd	nd	1.44	5.25E-09
525581225	Endo-beta-1,4-xylanase	Y	GH10	nd	nd	nd	nd
525583278	Endo-beta-1,4-xylanase	Y	GH11	1.93	9.90E-20	1.18	1.70E-11
525586882	Endo-beta-1,4-xylanase	Y	GH10	1.58	9.90E-20	3.40	9.90E-20
525584853	Endo-beta-1,6-galactanase	Y	GH30	0.20	7.77E-16	0.01	0.0061322
525588065	Exo-beta-1,3-galactanase	Y	GH43	2.88	2.70E-06	2.23	3.47E-06
525581916	Exo-beta-1,3-glucanase	Y	GH17	0.55	0.0025449	0.46	0.4394613

*nd* not detected; Y means with signal peptides; *GH* glycoside hydrolase.

### Expression and identification of pectinases and chitinases

This study identified nine pectin-degrading enzymes and four chitin-hydrolyzing enzymes (Additional file 4: Table S2). Only two pectinases and two chitinases were present in the secretome of AX. In the A secretome, 11 proteins involved in the degradation of pectin and chitin were identified, suggesting that A is the best substrate for the production of these enzymes. The number of proteins identified involved in the degradation of pectin and chitin was nine and seven in the X and G secretomes, respectively.

## Discussion

In a previous study, we found that *P. oxalicum* GZ-2 uses agricultural waste (corn stover) more efficiently than purified cellulose to produce cellulase. In contrast, *T. reesei* RUT-C30 was found to be more effective at secreting cellulolytic enzymes with purified cellulose as an inducer (data not published). As we know, cellulose is one of the most suitable substrates to induce cellulases for many fungi, especially for *T. reesei* RUT-C30 [1,28]. This interesting characteristic suggested that the induction and regulation mechanisms of *P. oxalicum* GZ-2 were different from those of *T. reesei* RUT-C30. The reasons why agricultural waste induces more enzymes for *P. oxalicum* GZ-2 are unclear. We hypothesize that xylan as the major component in the hemicellulose synergizes with cellulose to enhance lignocellulolytic enzyme induction. To validate this hypothesis and further evaluate the influence of the composition of complex substrates on enzyme induction, an artificial substrate containing a mixture of Avicel and xylan was designed to simulate plant biomass to study these questions.

As reported previously [29], the cellulase activity (FPase and CMCase) induced from *P. oxalicum* GZ-2 by the mixture of cellulose and xylan was significantly higher than that induced by purified cellulose. However, few reports have sought to explain why a mixture of xylan and cellulose enhances cellulase production. Contrary to *T. reesei* RUT-C30, purified cellulose is a poor inducer of cellulase production for *P. oxalicum* GZ-2. The result suggested that a difference potentially exists between these two strains in their production and regulation of cellulases. Another possible explanation is that *T. reesei* RUT-C30 is a mutant strain lacking repression by glucose, whereas *P. oxalicum* GZ-2 is not. In the A culture supernatant of strain GZ-2, a low concentration of glucose was detected during the fermentation period, which can repress the expression of cellulase. Wei *et al.* reported that a catabolite-repression-resistant mutant strain (*Penicillium decumbens* JU-A10) has a higher secretion capacity for cellulolytic enzymes than the wild-type strain *Penicillium decumbens* 114*-*2 [30]. Therefore, it is not surprising that *T. reesei* RUT-C30 produces cellulolytic enzymes more efficiently on cellulose medium*.*

The growth behavior of strain GZ-2 with various substrates was determined to evaluate the effect of fungal growth on enzymatic activity. The fungal cells of strain GZ-2 grew on the AX medium slightly faster than on the other substrates during the first three days (no significant difference at *P* <0.05), whereas the biomass of the A treatment was increased gradually and peaked at the end of fermentation. This may have happened because the low concentration reducing sugar was constantly supplemented as nutrition for growth from the enzymatic hydrolysis of A (data not shown). These results suggested that the increase in lignocellulolytic enzyme activity was not caused by better growth with the mixed carbon source.

The CMCase activity significantly increased when xylan was added to the cellulose medium; however, the culture supernatant of X did not display any CMCase activity. It was obvious that the increased CMCase activity was not due to xylan induction. We conjecture that xylan or its derivative products may activate regulatory factors that are able to enhance cellulase and hemicellulase expression. The xylanase regulator has been identified in *Trichoderma reesei* (*xyr1*) [31,32] and *Aspergillus niger* (*XlnR*) [33,34] as the main transcriptional activator regulating most xylanolytic and some cellulolytic enzyme genes. *XlnR* plays a key role in the xylan-triggered induction of cellulase and hemicellulase. For example, D-xylose and cellobiose trigger XlnR-dependent expression of xylanolytic and cellulolytic genes in *A. oryzae* [35]. Low cellulase activity was detected in the G culture, suggesting that G weakly induced cellulose-degrading enzymes. One cellulase protein (beta-glucosidase BGL1, 525581542) and six hemicellulases in the G secretome further confirmed the result of enzymatic activity. This result is not difficult to understand because low constitutive enzyme expression is necessary to initiate the formation of soluble sugars as inducers. Various monosaccharides and disaccharides derived from cellulosic biomass, such as D-xylose, cellobiose, gentiobiose, sophorose, and lactose, have been proposed to be cellulase inducers in different fungi [36-39].

Results of the quantitative polymerase chain reaction (q-PCR) for cellulolytic genes clearly showed that most genes (*egl1*, *egl2*, *egl*3, *sow*, and *cbh2*) were strongly expressed when AX was used as the substrate, explaining the high level of cellulase activity in the culture supernatant of AX. As we know, biomass-degrading enzymes are mainly regulated at the transcript level [40]. Significantly higher transcript levels of *egl1*, *egl2*, and *egl3* were measured in the AX treatment, indicating that these enzymes were major proteins in the control of CMCase activity. The expression level of *cbh2* is remarkably high compared with that of other genes. Consistent with the transcript result, the most abundant protein band (C3) observed on SDS-PAGE (Figure 2) and zymography gel (Figure 3A1) was identified as cellobiohydrolase, and this protein is a main cellulase in *Penicillium decumbens* 114*-*2 [41]. It is indicated that the enzyme cellobiohydrolase II (encoded by *cbh2*) was also the major cellulase in the strain GZ-2. The transcript level of *cbh2* was significantly higher in the AX culture among the four substrates, suggesting that the cellobiohydrolase II was strongly induced by AX. In most fungi, including *T. reesei* and *P. oxalicum*, cellobiohydrolase is the main cellulase in the cellulolytic system [41,42]. Unexpectedly, the best substrate for beta-glucosidase production was X instead of A or AX, and this was confirmed by the high transcript level of the beta-glucosidase gene *bgl* and the bright hydrolysis zone in-gel activity detection. Jørgensen *et al.* reported a similar result in which oat spelt xylan induced more beta-glucosidase than cellulose for *Penicillium persicinum* IBT 13226 [29]. For the cellobiohydrolase activity gel, a bright band was observed in lanes AX, suggesting that abundant cellobiohydrolase was present in the AX secretome. This result is consistent with the results of enzyme assay and gene expression.

A greater number of higher intensity protein bands were observed in the AX lane than in other lanes, indicating that the addition of xylan to the cellulose medium clearly changed the extracellular protein profiles. To compare the secretion patterns of (hemi)cellulolytic enzymes, zymogram gels incorporated with sodium carboxymethyl cellulose (CMC-Na) or beechwood xylan were run. Zymography is a powerful technology compared to the traditional colorimetric method because it not only can measure enzymatic activity but also visualize hydrolytic enzymes [43]. Using zymography, Liu *et al.* found that rice straw induces more protein bands with CMCase and xylanase activity than does Avicel [23]. The same number of bands with CMCase activity between lanes A and AX and the bigger pale-red hydrolysis zones of lane AX together indicated that the higher CMCase activity induced by AX was not due to a change of the protein species but instead to increased protein content. This result was further confirmed by the LC-MS/MS result that the same number of cellulases was identified in the A and AX secretomes. No CMCase activity was detected using the 3,5-dinitrosalicylic acid (DNS) method in the G or X culture supernatant, but some CMCase-active bands were observed in the zymogram. This result suggested that zymography is more sensitive than the colorimetric method for detecting CMCase activity. Other proteins, such as alpha-amylase and glucoamylase, were also identified in the zymogram by MALDI-TOF-MS/MS. These proteins may really hydrolyze CMC-Na but may not be the desired proteins with degrading ability because the excised band may contain several proteins. Therefore, to accurately identify a single protein with CMCase or xylanase activity, one-dimensional zymography is not sufficient. Accordingly, two-dimensional zymography should be used [44].

It is well known that xylanase is usually induced by xylan polymers and that cellulose is a poor substrate for producing xylanase. For example, Hori *et al.* reported that the addition of xylan to cellulose medium significantly increased xylanase activity and GH10 xylanase production in the basidiomycete *Phanerochaete chrysosporium* [45]. As expected, adding cellulose to the xylan culture medium had little effect on the xylanase production of *T. reesei* RUT-C30 (Additional file 1: Figure S1). However, adding cellulose to the xylan culture medium (A:X =2:1) strongly enhanced the xylanase production of *P. oxalicum* GZ-2. That the highest xylanase activity was induced by the AX substrate was further confirmed by sensitive zymography analysis. However, the xylanase activity was always low during fermentation on cellulose medium, suggesting that the increased xylanase activity was not directly caused by cellulose induction. It suggests that positive synergistic effects exist in enzyme induction between A and X when *P. oxalicum* GZ-2 is grown on a complex substrate. The significantly higher expression levels of xylanase genes (*xyl3* and *xyl4*) induced by AX suggest that two xylanases (encoded by *xyl3* and *xyl4*) are the reason for the increase in xylanase activity in the AX culture. The protein band (X10) showed the maximal hydrolysis zone (Figure 3A2), suggesting that it was the most abundant protein in the xylanase zymogram, and it was identified as GH11 xylanase II (encoded by *xyl2*). The high xylanase II abundance is in agreement with its gene (*xyl2*) being strongly expressed when X is used as a substrate. Although the content of GH11 xylanase II (encoded by *xyl2*) was dominant in the X secretome, the specific activity of xylanase II was found to be weaker than that of the other two xylanases (encoded by *xyl3* and *xyl4*) [46]. These results supported the idea that these two xylanases (encoded by *xyl3* and *xyl4*) are the key enzymes contributing to the high xylanase activity instead of xylanase II when cellulose is added to xylan medium. These results from the xylanase zymogram further confirmed that the addition of cellulose to xylan increased the abundance of hemicellulase species. Xylanase activity could be detected by neither colorimetry nor zymography when strain GZ-2 was grown on G, indicating the repression of these enzymes by glucose.

Interestingly, the secretome was significantly altered by the addition of xylan to the cellulose medium. In particular, the proportion of cellulases and hemicellulases was almost twofold higher in the AX secretome than in the others, which may be an important reason why lignocellulolytic enzyme production was enhanced when AX was used as the substrate. Three major cellulases, namely endo-1,4-beta-glucanase, exo-1,4-beta-glucanase, and beta-glucosidase, were also expressed in the A and X secretomes. However, the swollenin protein (525580909), which disrupts crystalline cellulose to enhance cellulosic substrate hydrolysis, and GH7 cellobiohydrolase (525584244) were only present in the AX secretome. A similar result was reported by Gómez-Mendoza *et al.*, in which the expansin-like protein (like the swollenin protein) only existed in the surgarcane bagasse secretome among four secretomes (glucose, CMC, xylan, and surgarcane bagasse) of *Trichoderma harzianum* [47]. It is noteworthy that many hemicellulases were secreted when strain GZ-2 was grown on the medium using A as the carbon source. However, the hemicellulase activity (xylanase and beta-xylosidase) was maintained at a low level (Figure 1), indicating that cellulose is a poor inducer of hemicellulase production. This phenomenon is normal because the same results have been found in other fungi such as *Postia placenta* [48], *Aspergillus fumigatus* [23], and *Trichoderma harzianum* [47]. The putative beta-1,3-1,4-glucanase (525581794), cellobiohydrolase Cel6A (525584431), and putative GH61 cellulose monooxygenase (525585914) were significantly upregulated in the AX treatment compared to the A treatment according to the AX/A ratio. These results indicated that more diverse functional protein species and higher expression of biomass-degrading proteins lead to the greater lignocellulolytic enzyme activity from the addition of xylan to cellulose cultures. The Bray-Curtis similarity indices, dendrogram analyses, and diversity indices together demonstrated that the secretome produced by *P. oxalicum* GZ-2 strictly depended on the substrate and that strain GZ-2 directionally changed the proportion of lignocellulolytic enzymes in its secretome according to the component of the substrate to promote subsistence on the complex substrate. Hori *et al.* studied the effects of xylan on the secretome of the basidiomycete *Phanerochaete chrysosporium* cultivated on cellulose medium using two-dimensional electrophoresis [45]. These authors found a similar result in that the addition of xylan to cellulose cultures significantly increased the xylanase and Avicelase activities. However, the expression of endo-beta-1,4-glucanase, beta-glucosidase, and cellobiohydrolase did not significantly increase in the mixed xylan and cellulose culture of *P. chrysosporium*. Furthermore, these authors’ studies are mainly focused on xylan-degrading enzymes, but less work was conducted to investigate the cellulase effect. Gómez-Mendoza *et al.* comparatively studied the secretomes of *Trichoderma harzianum* on CMC, xylan, and sugarcane bagasse [47]. Although the sugarcane bagasse-induced secretome from *Trichoderma harzianum* displayed the highest cellulolytic and xylanolytic activities, it did not correspond to greater proteome complexity because the cellulose-induced secretome was even more diverse.

Many studies have demonstrated that xylan is an effective inducer of hemicellulases [12,47,49,50]. In this study, up to five genetically different putative endo-beta-1,4-xylanases (two GH 10, two GH 11, and one GH 30) were detected in the X secretome, and four xylanases were observed in the AX and A secretomes, respectively. Unexpectedly, the proportion of hemicellulases (21.8%) in the AX treatment was increased significantly compared to that in the X (12.1%) or A (7.6%) treatment. Although more xylanases were induced by X, AX induced more other hemicellulases such as alpha-L-arabinofuranosidase and acetyl xylan esterase than X. These results indicated that these proteins have contributed to the significant increase of the xylanase activity. Liu *et al.* identified six genetically different endo-beta-1,4-xylanases in the *Penicillium decumbens* 114*-*2 secretome induced by a mixed medium (CW) consisting of cellulose plus wheat bran [41]. Beta-xylosidase was not detected in the *P. decumbens* 114*-*2 secretome of CW, although five genes were detected in the transcriptome. In this study, two beta-xylosidases were detected in each of the A, X, and AX secretomes, whereas only one was induced by G. These results suggest that *P. oxalicum* GZ-2 has more advantages in the degradation of hemicelluloses than does *P. decumbens* 114*-*2. Thus, many biomass-degrading enzymes are identified in the secretome, suggesting the potential of *P. oxalicum* GZ-2 as a versatile cell factory for the production of extracellular enzymes. Further works on molecular regulations are needed in order to understand the mechanisms of lignocellulolytic enzyme production under the induction of complex substrates.

## Conclusions

In lignocellulolytic enzyme production, cellulose and xylan have positively synergistic effects and they play an important role in the induction of highly efficient lignocellulolytic enzymes. More diverse functional protein species and the higher expression of certain enzymes are the primary contributors to the positively synergistic effect that results in greater enzymatic activity. The composition of the secretome of *P. oxalicum* GZ-2 strictly depended on the nature and component of the substrate. The fungus GZ-2 changed the proportion of lignocellulolytic enzymes in its secretome according to the type of substrate. These results were able to successfully explain the more highly efficient enzyme production induced by a complex substrate.

## Materials and methods

### Microorganisms, growth conditions, and secretome extraction

The *P. oxalicum* GZ-2 used in this study was isolated and identified as previously reported [12] and has been deposited into the China General Microbiological Culture Collection Center (CGMCC 7527). *T. reesei* RUT-C30 (ATCC 56765) was kindly provided by Irina S. Druzhinina (Vienna University of Technology, Vienna). The two strains were grown on potato dextrose medium at 30°C for 6 to 7 days. Conidia were harvested and made into a suspension at a concentration of 1 × 10^7^ conidia/mL. For secretome production, a basal medium (2.0 g of KH_2_PO_4_, 1.4 g of (NH_4_)_2_SO_4_, 1.0 g of tryptone, 0.3 g of urea, 0.4 g of CaCl_2_^.^2H_2_O, 0.3 g of MgSO_4_^.^7H_2_O, 7.5 mg of FeSO_4_^.^7H_2_O, 2.0 mg of MnSO_4_^.^H_2_O, 2.0 mg of ZnSO_4_, and 3.0 mg of CoCl_2_ in 1,000 mL of water, pH 5.0) was supplemented with 1% (w/v) cellulose (Avicel PH-101, Sigma, treatment A), xylan (beechwood xylan, Sigma, treatment X), a mixture of cellulose and xylan (cellulose: xylan =2:1, treatment AX), or glucose (treatment G). The ratio of A:X =2:1 was chosen to reflect the cellulose and hemicellulose composition of the majority of agricultural residues. An aliquot of 100 mL of supplemented medium was placed in a 500-mL Erlenmeyer flask, sterilized at 115°C for 30 min, inoculated with 1 mL of conidia suspension (1 × 10^7^ conidia/mL), and incubated at 30°C for 7 days (170 rpm). Each treatment was replicated three times. The fermented broths were centrifuged at 12,587 × g at 4°C for 20 min, the precipitated material was discarded, and the supernatant was filtered through a 0.22-μm membrane and further concentrated by freeze-drying. One hundred micrograms of the dried powder was dissolved in 10 mL of 50 mM Tris-HCl buffer (pH 8.0) and ultrafiltered through a 10-kDa molecular weight cut-off (MWCO) membrane (Sartorius, Göttingen, Germany). The total protein content in the ultrafiltered solution was determined using a MicroBCA protein assay kit (Dingguo Changsheng Biotechnology Co., Ltd., Beijing, China) with bovine serum albumin as the standard. The protein solution was stored at -80°C for later proteomic analysis.

### Lignocellulolytic enzyme activity and biomass assays

During fermentation, 2-mL samples were collected at regular intervals for lignocellulolytic enzyme activity assays. The filter paper (FPase) and endoglucanase (CMCase) activities were determined as described by Ghose [51] using Whatman grade 1 filter paper (1.0 × 6.0 cm) and 1% (w/v) sodium carboxymethyl cellulose (CMC-Na, Sigma, USA) as substrates, respectively. The xylanase activity was determined at 50°C for 10 min using beechwood xylan (1%, w/v, Sigma, USA) as the substrate according to Bailey *et al.* [52]. The reducing sugars released by the enzymatic reaction were determined using the DNS method [53] with glucose or xylose as the standard. One international unit (IU) of enzyme activity was defined as the amount of enzyme releasing 1 μmol of reducing sugar per minute under the assay conditions.

The beta-glucosidase, beta-xylosidase, and cellobiohydrolase activities were measured using 10 mM *p*-nitrophenyl-beta-D-glucopyranoside, *p*-nitrophenyl-beta-D-xylopyranoside, and *p*-nitrophenyl beta-D-cellobioside as substrates according to Parry *et al.* [54]. The reaction was subsequently terminated by adding 100 μL of 2 M Na_2_CO_3_. A Multi-detection Microplate Reader (SpectraMax M5, Molecular Devices, Sunnyvale, CA, USA) was used to read the absorbance at 405 nm. One unit of activity was defined as the amount of enzyme that was required to release 1 μmol of nitrophenol per minute.

A separate fermentation experiment was conducted to determine the fungal biomass. The entire fermented suspension was collected (X and G), filtered through dried filter paper, washed with MilliQ water (Millipore, Bedford, MA, USA) three times, and dried at 75°C to constant weight. The mycelial weight (A or AX) was calculated as the difference between the total dry weight of the solids (mycelium and residual cellulose) and that of the residual cellulose. The content of residual cellulose was determined according to Ahamed and Vermette [55].

### Protein profiles analysis using zymography and MALDI-TOF-MS/MS

Proteins in the ultrafiltered solution were profiled using SDS-PAGE (11% (w/v) polyacrylamide gel with a 5% stacking gel) as described by Laemmli [56] by loading approximately 100 μg of protein. The endoglucanase and xylanase activities were analyzed by zymogram according to Peterson *et al.* [57]. To do this, the polyacrylamide gel was incorporated with 1% CMC and xylan as substrates [23]. Both the SDS-PAGE and zymogram gels were run in the Mini-Protean II system (Bio-Rad) for 120 to 180 min at 120 V. The zymogram gel was stained with 0.1% (w/v) Congo red solution for 30 min followed by destaining with l M NaCl. To identify the proteins, the protein spots of interest were excised and in-gel digested with trypsin according to Liu *et al.* [23]. The digested proteins were identified using Bruker ultrafleXtreme MALDI-TOF-MS/MS (Bruker Daltonics, Karlsruhe, Germany). The protein candidates were searched in a proteome database of *Penicillium oxalicum* 114*-*2 that was downloaded from the National Center for Biotechnology Information (NCBI) database [58] using Mascot (Matrix Science, London, UK). The search parameters were set as follows: taxonomy fungi, enzyme trypsin, allow up to one missed cleavage, carbamidomethylation of cysteines as fixed modification, oxidation of methionine as variable modification, peptide mass tolerance of 120 ppm, and MS/MS tolerance of 0.6 Da. The identification of the proteins was considered positive when the Mascot score was *P* <0.05.

### RNA extraction and real-time quantitative PCR

For total RNA extraction, fresh mycelia of *P. oxalicum* GZ-2 induced under different carbon sources at 30°C for 48 h were ground in liquid nitrogen and suspended in Trizol reagent (Invitrogen, Carlsbad, CA, USA). The total RNA was extracted following the manufacturer’s protocol. Reverse transcription (RT) was performed using the PrimeScript™ RT reagent Kit and the gDNA Eraser Kit (Takara, Dalian, China). The RNA concentration was determined at 260 nm using a NanoDrop ND-2000 (Thermo Fisher Scientific, Wilmington, DE).

To determine the expression of lignocellulose-degrading genes, real-time quantitative PCR (q-PCR) was performed using the primers listed in Additional file 7: Table S4. Seven known genes encoding cellulose-degrading enzymes were studied in this work, namely three endoglucanase genes (*egl1*, KF233750; *egl2*, KF233751; and *egl3*, KF233752), one beta-glucosidase gene (*bgl*, KF233746), one swollenin gene (*sow*, KF233754), and two cellobiohydrolase genes (*cbh1*, KF233748; and *cbh2*, KF233749). Seven known genes encoding hemicellulose-degrading enzymes were selected, including four xylanase genes (*xyl1*, KF233755; *xyl2*, KF233756; *xyl3*, KF233757; and *xyl4*, KF233758), one beta-xylosidase gene (*b-x*, KF233747), one alpha-L-arabinofuranosidase gene (*arf*, KF233745), and one beta-1-4-mannanase gene (*ma*, KF233753). The gene expression copy number was calculated using a standard curve for each gene as described by Lee *et al.* [59]. The transcript number of the actin gene was quantified as an internal standard using the following primers: actin-F (CTCCATCCAGGCCGTTCTG) and actin-R (CATGAGGTAGTCGGTCAAGTCAC).

### Protein digestion, peptide extraction, and mass spectrometric analysis

Equal amounts of protein (2.0 mg) from each experimental condition were denatured in 1 mL of 8 M urea and reduced in 5 mM dithiothreitol (DTT) in 50 mM Tris-HCl (pH 8.0) at 95°C for 20 min. After cooling to room temperature, the protein was alkylated in 25 mM iodoacetamide (IAA) for 45 min in the darkness at room temperature. The final products were digested by adding 2% sequencing-grade trypsin (Promega, Madison, WI, USA) in urea (1.0 M)-NH_4_HCO_2_ (50 mM) (pH 7.8) at 37°C for 18 h. The peptide mixtures after digestion were lyophilized, desalted by Empore C18-SD disk cartridge (7 mm/3 mL, 3 M, Chrom Tech, Apple Valley, MN, USA) and further dried in a vacuum centrifuge.

The dried peptides were resuspended in 200 μL of 0.1% formic acid and separated by an Acclaim PepMap 100 column (C18, 3 μm, 100 Å) (Dionex, Sunnyvale, CA, USA) capillary with a 15-cm bed length using an UltiMate 3000 nano-HPLC (Thermo Fisher Scientific, San Jose, CA, USA) at a flow rate of 300 nL/min linked to an LTQ Orbitrap XL mass spectrometer (Thermo Scientific, San Jose, CA, USA). Two solvents, A (0.1% formic acid) and B (aqueous 80% acetonitrile in 0.08% formic acid), were used to elute the peptides from the nanocolumn. The gradient elutions were achieved using 5 to 40% of solvent B for 32 min, 40 to 95% B for 19 min, and maintained at 95% B for 9 min, with a total run time of 60 min. The electrospray voltage and the temperature of the ion transfer capillary were 2.2 kV and 200°C, respectively. The LTQ Orbitrap XL mass spectrometer was run in data-dependent acquisition mode using Xcalibur 2.2 software (Thermo Scientific) using the positive ion mode for data acquisition. Full-scan MS spectra (from m/z 350 to 1800) were acquired in the Orbitrap with a resolution of 60,000. The 10 most intense precursor ions greater than the threshold of 500 counts were selected for collision-induced fragmentation in the linear ion trap at a normalized collision energy of 35%. Dynamic exclusion was employed within 60 s to prevent the repetitive selection of peptides. A total of four technical replications were obtained for each biological replicate.

### Mass spectrometric data search and label-free quantitative analysis

All the MS/MS spectra were matched to specific proteins by searching against the FASTA proteome database of *Penicillium oxalicum* 114*-*2 that was downloaded from the NCBI database [58] using Proteome Discoverer software 1.3 (Thermo Scientific). Oxidation (M) was set as the dynamic modification; carbamidomethylation (C) was used as the static modification. The search results were filtered by a false discovery rate of 0.05 using a decoy database search. Protein identifications were accepted at >95% probability and contained at least one uniquely matched peptide. The signal peptide sequences were analyzed using the signal peptide prediction program SignalP version 4.1 [60]. The molecular mass and isoelectric point values were theoretical values obtained from the Compute pI/Mw tool [61] according to predicted amino acid sequences. PRIMER software (version 5.2.8, Plymouth Routines In Multivariate Ecological Research, PRIMER-E Ltd, Plymouth, UK) was used to analyze the Bray-Curtis similarity indices. The Bray-Curtis similarity indices were determined based on the detection or non-detection of a protein that was identified by LC-MS/MS in the secretome. A dendrogram was also generated using the PRIMER software.

For the quantitative analysis, SIEVE (Version 2.0, Thermo Fisher Scientific), a commercial label-free quantification package, was used to compare the relative abundance of proteins between the different carbon source treatments. Four raw MS files from each treatment were analyzed using the SIEVE software according to Katz *et al.* [62]. The experimental workflow of SIEVE is described as follows. First, align the chromatographic peaks that were detected by MS. Second, develop frames on all the parent ions that were scanned by MS/MS. Third, compare the area of the chromatographic peak of each sample within a frame and determine the ratios between two sample groups in a frame. Finally, identify all the frames with an MS/MS scan by importing the SEQUEST search results.

### Statistical analysis

The experiments were carried out in triplicate, and the results were subjected to Tukey’s HSD test for three independent samples at a 5% or 1% level of significance (*P* ≤0.05 or 0.01). All of the statistical analyses were performed using SPSS version 19.0 (SPSS Institute Inc., Cary, NC) and Microsoft office excel 2010. The LC-MS/MS alignment results are listed in Additional file 8.

## Additional files

Additional file 1: Figure S1 Lignocellulolytic enzyme activities in the culture supernatant and fungal biomass of *T. reesei* RUT-C30 in the presence of different substrates for 6 days at 30°C. The activities of FPase, CMCase, xylanase, and biomass are listed in A, B, C, and D, respectively. The error bars indicate the standard deviation of three replicates. Values in different treatments followed by the same letter are not significantly different according to Tukey’s honest significant difference test (*P* <0.05). Additional file 2: Figure S2 Fungal biomass (A) and protein concentration (B) analysis of *P. oxalicum* GZ-2 in the presence of various substrates during submerged fermentation for 7 days at 30°C. The error bars indicate the standard deviation of three replicates. Additional file 3: Table S1 The common proteins identified in all four treatments. Additional file 4: Table S2 The identified proteins in secretomes induced by various substrates. Additional file 5: Figure S3 Distribution of the identified proteins according to molecular mass and isoelectric point. Both the molecular mass and isoelectric point are theoretical values obtained from the Compute pI/Mw tool according to predicted amino acid sequences. Additional file 6: Table S3 The Simpson and Shannon-Wiener indices. Additional file 7: Table S4 Primers used for q-PCR in this study. Additional file 8: Table S5 The LC-MS/MS identification results.

## Abbreviations

A: Avicel
AX: mixture of Avicel and xylan
CBH: cellobiohydrolase
CMCase: carboxymethyl cellulase or endoglucanase
CMC-Na: sodium carboxymethyl cellulose
D index: Simpson diversity index
DNS: 3,5-dinitrosalicylic acid
DTT: dithiothreitol
FPase: filter paper activity
G: glucose
GH: glycoside hydrolase
H index: Shannon-Wiener index
LC-MS/MS: liquid chromatography-tandem mass spectrometry
MALDI-TOF-MS/MS: matrix-assisted laser desorption/ionization time-of-flight tandem mass spectrometry
MWCO: molecular weight cut-off
q-PCR: real-time quantitative polymerase chain reaction
X: xylan

## References

[1] Martinez D, Berka RM, Henrissat B, Saloheimo M, Arvas M, Baker SE, Chapman J, Chertkov O, Coutinho PM, Cullen D (2008). Genome sequencing and analysis of the biomass-degrading fungus *Trichoderma reesei* (syn. *Hypocrea jecorina*). Nat Biotechnol.

[2] Merino ST, Cherry J, Lisbeth O (2007). Progress and challenges in enzyme development for biomass utilization. Biofuels.

[3] Marjamaa K, Toth K, Bromann PA, Szakacs G, Kruus K (2013). Novel *Penicillium* cellulases for total hydrolysis of lignocellulosics. Enzyme Microb Technol.

[4] Liming X, Xueliang S (2004). High-yield cellulase production by *Trichoderma reesei* ZU-02 on corn cob residue. Bioresour Technol.

[5] Fang X, Yano S, Inoue H, Sawayama S (2008). Lactose enhances cellulase production by the filamentous fungus *Acremonium cellulolyticus*. J Biosci Bioeng.

[6] Mandels M, Reese ET: **Induction of cellulase in fungi by cellobiose.** *J Bacteriol* 1960, **79:**816. 10.1128/jb.79.6.816-826.1960 PMC278786 14420566

[7] Sternberg D, Mandels GR (1979). Induction of cellulolytic enzymes in *Trichoderma reesei* by sophorose. J Bacteriol.

[8] Morikawa Y, Ohashi T, Mantani O, Okada H (1995). Cellulase induction by lactose in *Trichoderma reesei* PC-3-7. Appl Microbiol Biotechnol.

[9] Karaffa L, Fekete E, Gamauf C, Szentirmai A, Kubicek CP, Seiboth B (2006). D-Galactose induces cellulase gene expression in *Hypocrea jecorina* at low growth rates. Microbiology.

[10] Do Vale LH, Gómez‐Mendoza DP, Kim MS, Pandey A, Ricart CA, Edivaldo Filho XF, Sousa MV (2012). Secretome analysis of the fungus *Trichoderma harzianum* grown on cellulose. Proteomics.

[11] Royer JC, Nakas J (1990). Interrelationship of xylanase induction and cellulase induction of *Trichoderma longibrachiatum*. Appl Environ Microbiol.

[12] Liao H, Xu C, Tan S, Wei Z, Ling N, Yu G, Raza W, Zhang R, Shen Q, Xu Y (2012). Production and characterization of acidophilic xylanolytic enzymes from *Penicillium oxalicum* GZ-2. Bioresour Technol.

[13] Fujii T, Fang X, Inoue H, Murakami K, Sawayama S (2009). Enzymatic hydrolyzing performance of *Acremonium cellulolyticus* and *Trichoderma reesei* against three lignocellulosic materials. Biotechnol Biofuels.

[14] Rana V, Eckard AD, Teller P, Ahring BK (2014). On-site enzymes produced from *Trichoderma reesei* RUT-C30 and *Aspergillus saccharolyticus* for hydrolysis of wet exploded corn stover and loblolly pine. Bioresour Technol.

[15] Juhasz T, Szengyel Z, Reczey K, Siika-Aho M, Viikari L (2005). Characterization of cellulases and hemicellulases produced by *Trichoderma reesei* on various carbon sources. Process Biochem.

[16] Alriksson B, Rose SH, van Zyl WH, Sjöde A, Nilvebrant N-O, Jönsson LJ (2009). Cellulase production from spent lignocellulose hydrolysates by recombinant *Aspergillus niger*. Appl Environ Microbiol.

[17] Li J, Lin L, Li H, Tian C, Ma Y (2014). Transcriptional comparison of the filamentous fungus *Neurospora crassa* growing on three major monosaccharides D-glucose, D-xylose and L-arabinose. Biotechnol Biofuels.

[18] Jourdier E, Cohen C, Poughon L, Larroche C, Monot F, Chaabane FB (2013). Cellulase activity mapping of *Trichoderma reesei* cultivated in sugar mixtures under fed-batch conditions. Biotechnol Biofuels.

[19] Andersen MR, Vongsangnak W, Panagiotou G, Salazar MP, Lehmann L, Nielsen J (2008). A trispecies *Aspergillus* microarray: comparative transcriptomics of three *Aspergillus* species. Proc Natl Acad Sci U S A.

[20] Delmas S, Pullan ST, Gaddipati S, Kokolski M, Malla S, Blythe MJ, Ibbett R, Campbell M, Liddell S, Aboobaker A: **Uncovering the genome-wide transcriptional responses of the filamentous fungus*****Aspergillus niger*****to lignocellulose using RNA sequencing.** *PLoS Genet* 2012, **8:**e1002875. 10.1371/journal.pgen.1002875 PMC3415456 22912594

[21] Munster JMv, Daly P, Delmas S, Pullan ST, Blythe MJ, Malla S, Kokolski M, Noltorp E, Wennberg K, Fetherston R: **The role of carbon starvation in the induction of enzymes that degrade plant-derived carbohydrates in*****Aspergillus niger*****.** *Fungal Genet Biol* 2014. 10.1016/j.fgb.2014.04.006 PMC4217149 24792495

[22] De Souza WR, Maitan-Alfenas GP, de Gouvêa PF, Brown NA, Savoldi M, Battaglia E, Goldman MHS, de Vries RP, Goldman GH (2013). The influence of *Aspergillus niger* transcription factors AraR and XlnR in the gene expression during growth in d-xylose, l-arabinose and steam-exploded sugarcane bagasse. Fungal Genet Biol.

[23] Liu D, Li J, Zhao S, Zhang R, Wang M, Miao Y, Shen Y, Shen Q: **Secretome diversity and quantitative analysis of cellulolytic*****Aspergillus fumigatus*****Z5 in the presence of different carbon sources.** *Biotechnol Biofuels* 2013, **6:**149. 10.1186/1754-6834-6-149 PMC3853031 24131596

[24] Saykhedkar S, Ray A, Ayoubi-Canaan P, Hartson SD, Prade R, Mort AJ (2012). A time course analysis of the extracellular proteome of *Aspergillus nidulans* growing on sorghum stover. Biotechnol Biofuels.

[25] Horta MAC, Vicentini R, da Silva DP, Laborda P, Crucello A, Freitas S, Kuroshu RM, Polikarpov I, da Cruz Pradella JG, Souza AP: **Transcriptome profile of*****Trichoderma harzianum*****IOC-3844 induced by sugarcane bagasse.** *PLoS One* 2014, **9:**e88689. 10.1371/journal.pone.0088689 PMC3928278 24558413

[26] De Souza WR, de Gouvea PF, Savoldi M, Malavazi I, de Souza Bernardes LA, Goldman MHS, de Vries RP, de Castro Oliveira JV, Goldman GH (2011). Transcriptome analysis of *Aspergillus niger* grown on sugarcane bagasse. Biotechnol Biofuels.

[27] Adav SS, Li AA, Manavalan A, Punt P, Sze SK (2010). Quantitative iTRAQ secretome analysis of *Aspergillus niger* reveals novel hydrolytic enzymes. J Proteome Res.

[28] Alvira P, Gyalai‐Korpos M, Barta Z, Oliva JM, Réczey K, Ballesteros M (2013). Production and hydrolytic efficiency of enzymes from *Trichoderma reesei RUTC30* using steam pretreated wheat straw as carbon source. J Chem Technol Biotechnol.

[29] Jorgensen H, Morkeberg A, Krogh KBR, Olsson L (2005). Production of cellulases and hemicellulases by three *Penicillium* species: effect of substrate and evaluation of cellulase adsorption by capillary electrophoresis. Enzyme Microb Technol.

[30] Wei X, Zheng K, Chen M, Liu G, Li J, Lei Y, Qin Y, Qu Y (2011). Transcription analysis of lignocellulolytic enzymes of *Penicillium decumbens* 114*-*2 and its catabolite-repression-resistant mutant. C R Biol.

[31] Mach-Aigner AR, Pucher ME, Steiger MG, Bauer GE, Preis SJ, Mach RL (2008). Transcriptional regulation of xyr1, encoding the main regulator of the xylanolytic and cellulolytic enzyme system in *Hypocrea jecorina*. Appl Environ Microbiol.

[32] Furukawa T, Shida Y, Kitagami N, Mori K, Kato M, Kobayashi T, Okada H, Ogasawara W, Morikawa Y (2009). Identification of specific binding sites for XYR1, a transcriptional activator of cellulolytic and xylanolytic genes in *Trichoderma reesei*. Fungal Genet Biol.

[33] Gielkens MM, Dekkers E, Visser J, de Graaff LH (1999). Two cellobiohydrolase-encoding genes from *Aspergillus niger* require D-xylose and the xylanolytic transcriptional activator XlnR for their expression. Appl Environ Microbiol.

[34] Van Peij NN, Gielkens MM, de Vries RP, Visser J, de Graaff LH (1998). The transcriptional activator XlnR regulates both xylanolytic and endoglucanase gene expression in *Aspergillus niger*. Appl Environ Microbiol.

[35] Stricker AR, Steiger MG, Mach RL (2007). Xyr1 receives the lactose induction signal and regulates lactose metabolism in *Hypocrea jecorina*. FEBS Lett.

[36] Mach-Aigner AR, Pucher ME, Mach RL (2010). D-Xylose as a repressor or inducer of xylanase expression in *Hypocrea jecorina* (*Trichoderma reesei*). Appl Environ Microbiol.

[37] Marui J, Tanaka A, Mimura S, de Graaff LH, Visser J, Kitamoto N, Kato M, Kobayashi T, Tsukagoshi N (2002). A transcriptional activator, AoXlnR, controls the expression of genes encoding xylanolytic enzymes in *Aspergillus oryzae*. Fungal Genet Biol.

[38] Kurasawa T, Yachi M, Suto M, Kamagata Y, Takao S, Tomita F (1992). Induction of cellulase by gentiobiose and its sulfur-containing analog in *Penicillium purpurogenum*. Appl Environ Microbiol.

[39] Stricker AR, Grosstessner-Hain K, Würleitner E, Mach RL (2006). Xyr1 (xylanase regulator 1) regulates both the hydrolytic enzyme system and D-xylose metabolism in *Hypocrea jecorina*. Eukaryot Cell.

[40] Tani S, Kawaguchi T, Kobayashi T (2014). Complex regulation of hydrolytic enzyme genes for cellulosic biomass degradation in filamentous fungi. Appl Microbiol Biotechnol.

[41] Liu G, Zhang L, Wei X, Zou G, Qin Y, Ma L, Li J, Zheng H, Wang S, Wang C: **Genomic and secretomic analyses reveal unique features of the lignocellulolytic enzyme system of*****Penicillium decumbens*****.** *PLoS One* 2013, **8:**e55185. 10.1371/journal.pone.0055185 PMC3562324 23383313

[42] Ilmen M, Saloheimo A, Onnela M-L, Penttilä ME (1997). Regulation of cellulase gene expression in the filamentous fungus *Trichoderma reesei*. Appl Environ Microbiol.

[43] Vandooren J, Geurts N, Martens E, Van den Steen PE, Opdenakker G (2013). Zymography methods for visualizing hydrolytic enzymes. Nat Meth.

[44] Kim KH, Brown KM, Harris PV, Langston JA, Cherry JR (2007). A proteomics strategy to discover β-glucosidases from *Aspergillus fumigatus* with two-dimensional page in-gel activity assay and tandem mass spectrometry. J Proteome Res.

[45] Hori C, Igarashi K, Katayama A, Samejima M (2011). Effects of xylan and starch on secretome of the basidiomycete *Phanerochaete chrysosporium* grown on cellulose. FEMS Microbiol Lett.

[46] Liao H, Sun S, Wang P, Bi W, Tan S, Wei Z, Mei X, Liu D, Raza W, Shen Q (2014). A new acidophilic endo-β-1,4-xylanase from *Penicillium oxalicum*: cloning, purification, and insights into the influence of metal ions on xylanase activity. J Ind Microbiol Biotechnol.

[47] Gómez-Mendoza DP, Junqueira M, Do Vale LHF, Domont GB, Filho EXF, Sousa MVD, Ricart CAO (2014). Secretomic survey of *Trichoderma harzianum* grown on plant biomass substrates. J Proteome Res.

[48] Martinez D, Challacombe J, Morgenstern I, Hibbett D, Schmoll M, Kubicek CP, Ferreira P, Ruiz-Duenas FJ, Martinez AT, Kersten P (2009). Genome, transcriptome, and secretome analysis of wood decay fungus *Postia placenta* supports unique mechanisms of lignocellulose conversion. Proc Natl Acad Sci U S A.

[49] Bailey MJ, Buchert J, Viikari L (1993). Effect of pH on production of xylanase by *Trichoderma reesei* on xylan- and cellulose-based media. Appl Biochem Biotechnol.

[50] Chávez R, Bull P, Eyzaguirre J (2006). The xylanolytic enzyme system from the genus *Penicillium*. J Biotechnol.

[51] Ghose T (1987). Measurement of cellulase activities. Pure Appl Chem.

[52] Bailey MJ, Biely P, Poutanen K (1992). Interlaboratory testing of methods for assay of xylanase activity. J Biotechnol.

[53] Miller GL (1959). Use of dinitrosalicylic acid reagent for determination of reducing sugar. Anal Chem.

[54] Parry NJ, Beever DE, Owen E, Vandenberghe I, Van Beeumen J, Bhat MK (2001). Biochemical characterization and mechanism of action of a thermostable beta-glucosidase purified from *Thermoascus aurantiacus*. Biochem J.

[55] Ahamed A, Vermette P (2008). Culture-based strategies to enhance cellulase enzyme production from *Trichoderma reesei* RUT-C30 in bioreactor culture conditions. Biochem Eng J.

[56] Laemmli UK (1970). Cleavage of structural proteins during the assembly of the head of bacteriophage T4. Nature.

[57] Peterson R, Grinyer J, Nevalainen H (2011). Extracellular hydrolase profiles of fungi isolated from koala faeces invite biotechnological interest. Mycol Prog.

[58] **The National Center for Biotechnology Information.** [https://www.ncbi.nlm.nih.gov]

[59] Lee C, Lee S, Shin SG, Hwang S (2008). Real-time PCR determination of rRNA gene copy number: absolute and relative quantification assays with *Escherichia coli*. Appl Microbiol Biotechnol.

[60] Petersen TN, Brunak S, von Heijne G, Nielsen H (2011). SignalP 4.0: discriminating signal peptides from transmembrane regions. Nat Meth.

[61] **Compute pI/Mw tool.** [http://web.expasy.org/compute_pi/]

[62] Katz E, Fon M, Eigenheer RA, Phinney BS, Fass JN, Lin D, Sadka A, Blumwald E: **A label-free differential quantitative mass spectrometry method for the characterization and identification of protein changes during citrus fruit development.** *Proteome Sci* 2010, **8:**68. 10.1186/1477-5956-8-68 PMC3017515 21162737

