# Supplementary figures and images for: Insights into high-efficiency lignocellulolytic enzyme production by Penicillium oxalicum GZ-2 induced by a complex substrate
Source: Biotechnol Biofuels. 2014 Nov 18;7:162. doi: 10.1186/s13068-014-0162-2 (PMC4239378; doi:10.1186/s13068-014-0162-2)

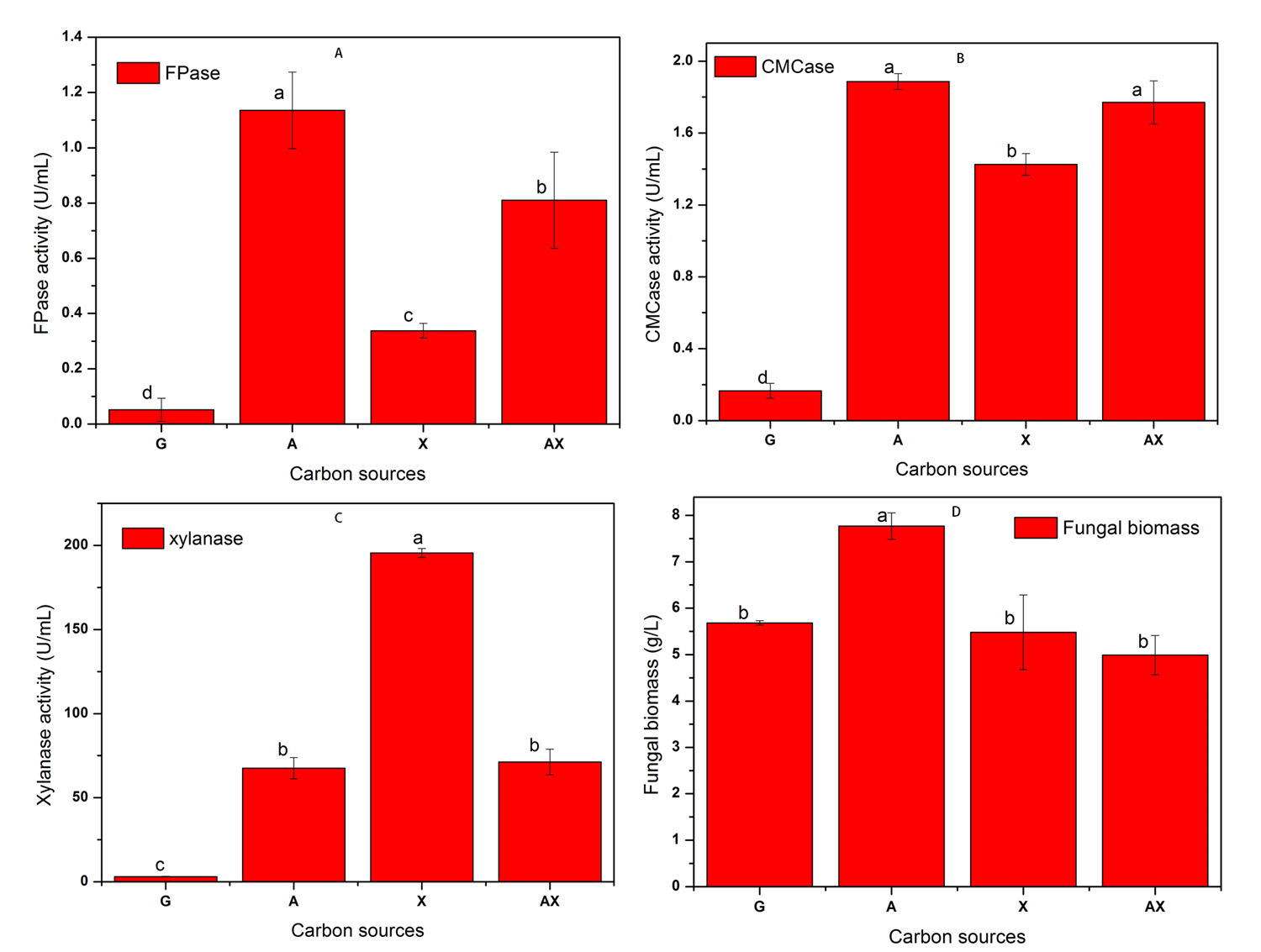

Supplement: Additional file 1: Figure S1 — Lignocellulolytic enzyme activities in the culture supernatant and fungal biomass of T. reesei RUT-C30 in the presence of different substrates for 6 days at 30°C. The activities of FPase, CMCase, xylanase, and biomass are listed in A, B, C, and D, respectively. The error bars indicate the standard deviation of three replicates. Values in different treatments followed by the same letter are not significantly different according to Tukey’s honest significant difference test (P <0.05). [file 13068_2014_162_MOESM1_ESM.tiff]

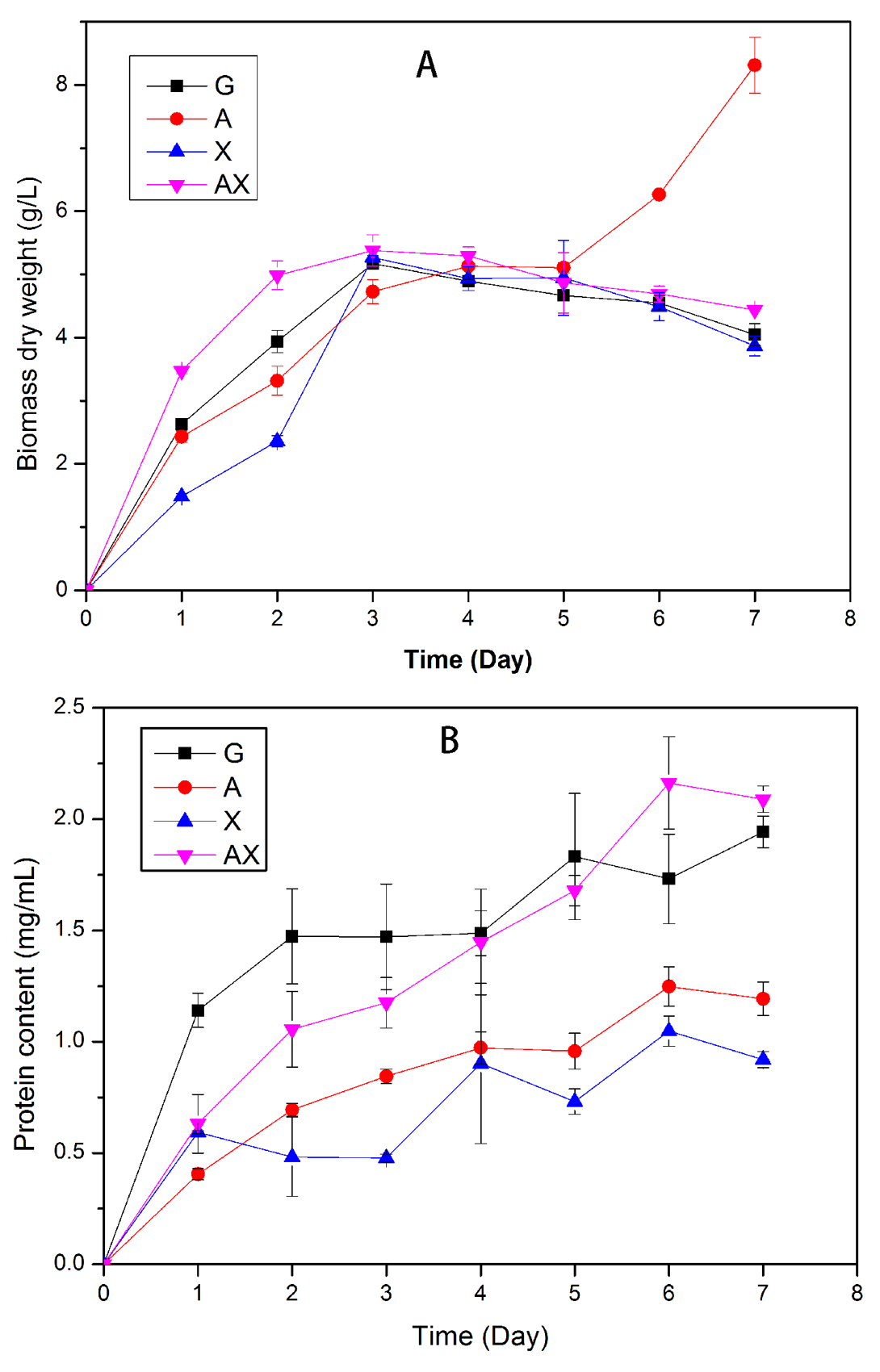

Supplement: Additional file 2: Figure S2 — Fungal biomass (A) and protein concentration (B) analysis of P. oxalicum GZ-2 in the presence of various substrates during submerged fermentation for 7 days at 30°C. The error bars indicate the standard deviation of three replicates. [file 13068_2014_162_MOESM2_ESM.tiff]
